# Mechanistic basis for protection of differentiated SH-SY5Y cells by oryzanol-rich fraction against hydrogen peroxide-induced neurotoxicity

**DOI:** 10.1186/1472-6882-14-467

**Published:** 2014-12-05

**Authors:** Norsharina Ismail, Maznah Ismail, Mustapha Umar Imam, Nur Hanisah Azmi, Siti Farhana Fathy, Jhi Biau Foo, Muhammad Firdaus Abu Bakar

**Affiliations:** Nutricosmeceuticals and Nutrigenomics Programme, Laboratory of Molecular Biomedicine, Institute of Bioscience, Universiti Putra Malaysia, Serdang, Selangor 43400 Malaysia; Department of Nutrition and Dietetics, Faculty of Medicine and Health Sciences, Universiti Putra Malaysia, Serdang, Selangor 43400 Malaysia

**Keywords:** Neuroprotective, Rice bran, Oryzanol-rich fraction, Hydrogen peroxide, Supercritical fluid extraction system, Multiplex GeXP, SH-SY5Y cells

## Abstract

**Background:**

Apoptosis is often the end result of oxidative damage to neurons. Due to shared pathways between oxidative stress, apoptosis and antioxidant defence systems, an oxidative insult could end up causing cellular apoptosis or survival depending on the severity of the insult and cellular responses. Plant bioresources have received close attention in recent years for their potential role in regulating the pathways involved in apoptosis and oxidative stress in favour of cell survival. Rice bran is a bioactive-rich by-product of rice milling process. It possesses antioxidant properties, making it a promising source of antioxidants that could potentially prevent oxidative stress-induced neurodegenerative diseases.

**Methods:**

Thus, the present study investigated the neuroprotective properties of oryzanol-rich fraction (ORF) against hydrogen peroxide (H_2_O_2_)-induced neurotoxicity in differentiated human neuroblastoma SH-SY5Y cells. ORF was extracted from rice bran using a green technology platform, supercritical fluid extraction system. Furthermore, its effects on cell viability, morphological changes, cell cycle, and apoptosis were evaluated. The underlying transcriptomic changes involved in regulation of oxidative stress, apoptosis and antioxidant defence systems were equally studied.

**Results:**

ORF protected differentiated SH-SY5Y cells against H_2_O_2_-induced neurotoxicity through preserving the mitochondrial metabolic enzyme activities, thus reducing apoptosis. The mechanistic basis for the neuroprotective effects of ORF included upregulation of antioxidant genes (catalase, SOD 1 and SOD 2), downregulation of pro-apoptotic genes (JNK, TNF, ING3, BAK1, BAX, p21 and caspase-9), and upregulation of anti-apoptotic genes (ERK1/2, AKT1 and NF-Kβ).

**Conclusion:**

These findings suggest ORF may be an effective antioxidant that could prevent oxidative stress-induced neurodegenerative disorders.

## Background

Antioxidant defense systems scavenge reactive oxygen species (ROS) in biological systems as a way to prevent build-up of their levels beyond physiologically acceptable limits. At a certain threshold, however, cells become unable to remove excess ROS, leading to oxidative stress, which is linked to the degenerative processes of aging as well as pathogenesis of many diseases [1]. Cellular response to oxidative stress is dependent on the type of cell. Responses to neuronal oxidative stress are particularly interesting because of their contrasting nature in comparison to other cells. Furthermore, with increasing longevity due to better health care systems, and the delicate nature of the nervous system, considerable interest has grown in factors linked to neurodegenerative diseases in a bid to improve quality of life of the elderly.

Superoxide anions, hydrogen peroxide (H_2_O_2_) and the hydroxyl radical have been indicated as potent mediators of neuronal oxidative stress [2]. They elicit a complex cascade of events that may eventually remove the initiating stimulus or result in apoptotic death of the cells, depending on the severity of the damage [1]. Mitochondria have been suggested as early targets of oxidative damage, in which cause damage leads to cytochrome c release through a process closely regulated by the Bcl-2 family proteins (Bcl-2, Bax and Bid). Cytochrome c in conjunction with Apaf-1 then activates the caspases, which cleave DNA repair enzymes including PARP, eventually leading to cellular damage and apoptosis [3]. Interestingly, cell survival signaling pathways are closely linked to those that end up in apoptosis. Notably, the major signaling pathways in response to oxidative stress insults include mitogen-activated protein kinases (MAPKs), Akt pathway and nuclear factor-kβ (NF-κβ) signaling. MAPKs encompass a large number of serine/threonine kinases involved in regulating cellular processes including proliferation, differentiation, stress adaptation, and apoptosis. These include the extracellular signal-regulated kinases (ERK), the c-Jun N-terminal kinases (JNK), and the p38 kinases. The ERK pathway is linked to the regulation of cell proliferation, while the JNK and p38 pathways are more strongly tied to stress [1].

Furthermore, Akt activation mediated through phosphatidylinositol-3 kinase (PI3K) pathway has been reported to inhibit apoptosis by inhibiting caspase-9 and Bad. ERK1/2 activation through active Ras and PKA has also been shown to block cytochrome c release through Bad-mediated Bcl-xL inhibition [3]. On the other hand, NF-κB also regulates inflammation, immune responses, control of cell division and apoptosis, and its manipulation is reported to be valuable in treating ischemic stroke, physical trauma to the brain or spinal cord, and neurodegenerative disorders including Alzheimer’s disease and Parkinson’s disease [4]. Overall, the modulation of these complex pathways constitutes an important avenue for therapeutic interventions aimed at limiting oxidative damage or attenuating its consequent effects [1].

In recent years, growing concerns of side effects associated with pharmacological agents have generated interest in the therapeutic potentials of plant bioresources. Rice bran is a by-product of the rice milling process, and was previously considered a waste. Now, it is known to contain fat, proteins and bioactives including γ-oryzanol (a mixture of ferulic acid esters of triterpene alcohols and sterols), tocols (tocopherols and tocotrienols) and unsaturated fatty acids [5–9], phytosterols, stanols and policosanols [10]. Its functional effects include antioxidant, anti-inflammatory, cholesterol-lowering and anti-diabetic, anti-cancer, anti-hypertensive and glucose metabolism [5, 11–15]. Simultaneous extraction and use of multiple bioactive compounds from plants have been reported to potentiate the effects of any one of the bioactives through synergy. Hence, our choice of oryzanol-rich fraction (ORF) in the current study was to maximize the benefits from the rice bran bioactives, while using a green technology, supercritical fluid extraction (SFE) system [16–23].

In the present study, extracted ORF was studied for its ability to regulate processes leading up to oxidative stress and apoptosis in differentiated SH-SY5Y cells.

## Methods

### Reagents

Rice bran samples were obtained from local milling company, Padiberas National Berhad (BERNAS) at Kuala Selangor, Malaysia. The human neuroblastoma SH-SY5Y cell line was obtained from American Type Culture Collection (Manassas, VA, USA). Minimum essential Eagle’s medium, Ham’s nutrient mixture F-12, fetal bovine serum and gentamicin were obtained from Sigma (St. Louis, MO, USA). Total RNA Isolation kit was obtained from RBC Bioscience Corp. (Taipei, Taiwan), GenomeLab™ GeXP Start Kit was purchased from Beckman Coulter Inc. (Miami, FL, USA), Magnesium chloride and DNA Taq polymerase were from Thermo Fisher Scientific (Pittsburgh, PA, USA).

### Extraction of ORF by SFE system

ORF was prepared using SFE system (Thar 1000 F, Thar Technologies, Inc., Pittsburgh, PA, USA). Briefly, 100 g of stabilized rice bran was placed into the SFE extraction vessel and extraction parameters were set at 600 bars pressure, temperature of 40°C and carbon dioxide flow rate of 30 g/min. ORF was collected from collection vessel when the ranges of pressure and temperature reached 100 – 300 bar and 40°C – 60°C, respectively.

### Cell culture

The human neuroblastoma SH-SY5Y cells were maintained in complete culture medium containing 1:1 mixture of Minimum essential Eagle's medium and Ham's nutrient mixture F-12, supplemented with 10% fetal bovine serum, 1% MEM non-essential amino acids and 50 μg/mL gentamicin. Cells were maintained at 37°C under 5% CO_2_/95% air.

### MTT assay

SH-SY5Y cells were seeded into 96-well culture plates at a density of 2 × 10^5^ cells/mL and allowed to attach. Then, 24 h after seeding, the cells were differentiated with retinoic acid (10 μM) for 6 days prior to treatment. The differentiated cells were then pretreated for 24 h with ORF prepared in serum-free medium at concentrations of 1, 10 and 100 μg/mL. The treated cells were then challenged with 250 μM H_2_O_2_ for 24 h, as reported in our previous publication [24]. MTT [3-(4,5-dimethylthiazol-2-yl)-2,5-diphenyl-tetrazolium bromide, Sigma, St. Louis, MO, USA] was added to the wells and allowed to incubate in the dark at 37°C for 4 h. The amount of MTT formazan product was determined by measuring absorbance using a Microplate reader (Opsys MR, Thermo Labsystems, Franklin, MA) at 570 nm.

### Acridine orange (AO)–propidium iodide (PI) double staining cell morphological assessment

Approximately 2 × 10^5^ cells/mL of SH-SY5Y were seeded into 6-well plate and allowed to attach. Then, 24 h after seeding, the cells were differentiated with retinoic acid (10 μM) for 6 days prior to treatment. The differentiated cells were then pretreated for 24 h with 100 μg/mL ORF prepared in serum-free medium, and subsequently exposed to 250 μM H_2_O_2_ for 24 h. The cells were then trypsinized and 10 *μ* L of the cell suspension was mixed with 10 *μ* L of AO (50 *μ* g/mL) and PI (50 *μ* g/mL) and placed on a glass slide. The cells were viewed under a fluorescence microscope (Leica, Germany).

### Cell cycle analysis

SH-SY5Y cells were seeded into 6-well plates at a density of 2 × 10^5^ cells/mL. The cells were differentiated with 10 μM retinoic acid for 6 days prior to treatment. The cells were pretreated with 100 μg/mL ORF for 24 h with subsequent exposure to 250 μM H_2_O_2_ for 24 h. The cells were harvested using 0.1% trypsin-EDTA, fixed in 70% ethanol and kept at -20°C overnight. After fixation, the pellets were washed with PBS to remove ethanol and further resuspended in 25 μL of RNAse, 50 μL of propidium iodide and 425 μL of PBS to make up the volume to 500 μL. After 30 min of incubation in the dark at 4°C, the DNA contents of the cells were analyzed using flow cytometer with Summit v4.3 software (Cyan ADP, Beckman Coulter, Brea, CA, USA).

### Annexin V-FITC and propidium iodide staining assay

SH-SY5Y cells were seeded in 6-well plates at a density of 2 × 10^5^ cells/mL. The cells were differentiated with 10 μM retinoic acid for 6 days prior to treatment. The cells were pretreated with 100 μg/mL ORF for 24 h followed by exposure to 250 μM H_2_O_2_ for another 24 h. The subsequent procedures were carried out according to the instructions provided by the manufacturer of APOPTEST-FITC kit (Beckman Coulter, Brea, CA, USA). Briefly, cells were harvested using 0.1% trypsin-EDTA and cell pellets were resuspended in ice-cold 1X binding buffer. One microliter of Annexin V-FITC solution and 5 μL of propidium iodide were added to 100 μL of the cell suspension. The tube was incubated on ice for 15 min in the dark followed by addition of 400 μL ice-cold 1X binding buffer and mixing gently. The samples were analyzed using flow cytometer with Summit software v4.3 (CyAN ADP, Beckman Coulter, Brea, CA, USA).

### GeXP multiplex gene expression analysis

#### RNA extraction

SH-SY5Y cells were seeded into 6-well plates at a density of 2 × 10^5^ cells/mL. The cells were differentiated with 10 μM retinoic acid for 6 days prior to treatment. The cells were pretreated with 100 μg/mL ORF for 24 h with subsequent exposure to 250 μM H_2_O_2_ for 24 h. Total RNA was extracted using Total RNA Isolation kit (RBC Bioscience Corp., Taiwan) according to the manufacturer’s protocol. RNA concentration was quantified using NanoDrop spectrophotometer (Thermo Scientific Nanodrop, NanoDrop Technologies, Wilmington, DE, USA), and ratios of A260/230 and A260/280 between 1.8 and 2.0 were used to indicate RNA of high purity.

#### Primer design

Nucleotide sequences of the genes of interest and housekeeping genes (Table 1) were obtained from National Center for Biotechnology Information GenBank Database, while the internal control (KanR) was supplied by Beckman Coulter Inc. (Miami, FL, USA). The specificity validation of the nucleotide sequences was performed using NCBI-nucleotide-BLAST. Additional 37 base pair of universal tag sequences were attached to each forward and reverse primers. Synthesis of primers was done by First Base Ltd. (Selangor, Malaysia) and diluted according to instructions from Beckman Coulter Inc (Miami, FL, USA).

**Table 1:** **Gene name, accession number, reverse and forward primer sequences used in GeXP multiplex gene expression analysis**

Gene name	Accession number	Primer sequences* with universal tags	
		Forward	Reverse
*Antioxidant genes*			
**Catalase**	NM_001752	*AGGTGACACTATAGAATA* GAAGTGCGGAGATTCAACACT	*GTACGACTCACTATAGGGA* ACACGGATGAACGCTAAGCT
**SOD 1**	NM_000454	*AGGTGACACTATAGAATATCA* TCAATTTCGAGCAGAAGG	*GTACGACTCACTATAGGGA* TGCTTTTTCATGGACCACC
**SOD 2**	NM_000636	*AGGTGACACTATAGAATA* CATCAAACGTGACTTTGGTTC	*GTACGACTCACTATAGGGA* CTCAGCATAACGATCGTGGTT
*Downstream apoptotic genes*			
**BAD**	NM_004322	*AGGTGACACTATAGAATA* CGGAGGATGAGTGACGAGTT	*GTACGACTCACTATAGGG* AGGAGTTTCGGGATGTGGAG
**TNF**	NM_000594	*AGGTGACACTATAGAATA* CTATCTGGGAGGGGTCTTCC	*GTACGACTCACTATAGGG* AATGTTCGTCCTCCTCACAGG
**ING3**	NM_198267	*AGGTGACACTATAGAATA* AGGTTCAGTTGGCAAACCAG	*GTACGACTCACTATAGGG* AAGCCAAAAGTGAGCATGTGTT
**BAK1**	BC004431	*AGGTGACACTATAGAATA* AGCCTGTTTGAGAGTGGCAT	*GTACGACTCACTATAGGG* AAGTGATGCAGCATGAAGTCG
**BAX**	BC014175	*AGGTGACACTATAGAATA* CCCTTTTGCTTCAGGGTTTC	*GTACGACTCACTATAGGG* ACAAAGTAGAAAAGGGCGACAA
**p21**	NM_000389	*AGGTGACACTATAGAATA* TTAGCAGCGGAACAAGGAGT	*GTACGACTCACTATAGGG* AAGCCGAGAGAAAACAGTCCA
**CAS-9**	NM_001229	*AGGTGACACTATAGAATA* GGGCTCACTCTGAAGACCTG	*GTACGACTCACTATAGGG* ATCTGGAAGCTGCTAAGAGCC
**BCL-2**	M14745	*AGGTGACACTATAGAATA* ACCACTAATTGCCAAGCACC	*GTACGACTCACTATAGGG* ATTTTCCATCCGTCTGCTCTT
*Upstream apoptotic genes*			
**ERK1/2**	NM_002745	*AGGTGACACTATAGAATA* GGAGCAGTATTACGACCCGA	*GTACGACTCACTATAGGGA* GATGTCTGAGCACGTCCAGT
**p53**	NM_001126117	*AGGTGACACTATAGAATA* GGGGAGCAGGGCTCA	*GTACGACTCACTATAGGGA* AAAATGGCAGGGGAGGG
**JNK**	NM_139046	*AGGTGACACTATAGAATA* CAGAAGCTCCACCACCAAAGAT	*GTACGACTCACTATAGGGA* GCCATTGATCACTGCTGCAC
**PARP1**	NM_001618	*AGGTGACACTATAGAATA* TATCGAGTCGAGTACGCCAA	*GTACGACTCACTATAGGGA* GTGTGGGACTTTTCCATCAAA
**AKT1**	NM_001014431	*AGGTGACACTATAGAATA* GAGGAGATGGACTTCCGGTC	*GTACGACTCACTATAGGGA* AGGATCTTCATGGCGTAGTAGC
**NF-kB**	NM_001077493	*AGGTGACACTATAGAATA* GCGGGCGTCTAAAATTCTG	*GTACGACTCACTATAGGG* ATTCCACGATCACCAGGTAGG
**p38**	NM_001315	*AGGTGACACTATAGAATA* TTCAGTCTTTGACTCAGATGCC	*GTACGACTCACTATAGGGA* GTCAGGCTTTTCCACTCATCT
*Housekeeping genes*			
**GAPDH** ^**a**^	NM_002046	*AGGTGACACTATAGAATA* AAGGTGAAGGTCGGAGTCAA	*GTACGACTCACTATAGGGA* GATCTCGCTCCTGGAAGATG
**Hyaluronidase** ^**a**^	AJ000099	*AGGTGACACTATAGAATA* CAGCAGTTCATGCTGGAGAC	*GTACGACTCACTATAGGGA* CCAGGTAGACAGACGGGAAG
**18 sRNA** ^**a**^	M10098	*AGGTGACACTATAGAATA* GGAGTGGAGCCTGCGGCTTAA	*GTACGACTCACTATAGGGA* TAGCATGCCAGAGTCTCGTT
**Actb** ^**a,#**^	NM_001101	*AGGTGACACTATAGAATA* GATCATTGCTCCTCCTGAGC	*GTACGACTCACTATAGGGA* AAAGCCATGCCAATCTCATC
**Kan(r)** ^**b**^	-	*AGGTGACACTATAGAATA* ATCATCAGCATTGCATTCGATTCCTGTTTG	*GTACGACTCACTATAGGGA* ATTCCGACTCGTCCAACATC

*Based on the *Homo sapien* gene sequences adopted from the National Center for Biotechnology Information GenBank Database.

^a^Housekeeping genes; ^b^Internal control; ^#^Normalization gene.

#### cDNA synthesis

The complementary DNA (cDNA) was synthesized using 50 ng/μL RNA of each sample. The reverse transcription (RT) reaction was performed according to GenomeLab™ GeXP Start Kit instructions (Beckman Coulter Inc., Miami, FL, USA): 1 μL of RNA sample, 4 μL of 5X RT buffer, 2 μL of RT multiplex reverse primers, 1 μL of KanR, 1 μL of reverse transcriptase and 11 μL of DNAse/RNase free water. cDNA was synthesized according to the reaction protocol: 48°C for 1 min, 42°C for 60 min, 95°C for 5 min and 4°C hold in XP Thermal Cycler (BIOER Technology, Hangzhou, China).

#### PCR amplification

PCR reactions were carried out using GeXP Start Kit (Beckman Coulter, Miami, FL, USA) consisting of cDNA sample taken from the RT reaction (9.3 μL each), 5X PCR buffer, 25 mM magnesium chloride, PCR multiplex forward primer and Thermo-Start DNA polymerase. Amplification was done in an XP Thermal Cycler (BIOER Technology, Hangzhou, China) using 95°C for 10 min, followed by 34 cycles of 94°C for 30 sec, 55°C for 30 sec, 70°C for 1 min and 4°C hold.

#### GeXP multiplex data analysis

The PCR product (l μL each) was mixed with 38.5 μL of sample loading solution and 0.5 μL of DNA Size Standard-400 (Beckman Coulter Inc., Miami, FL, USA). The PCR products were then separated in the GenomeLab GeXP Genetic Analysis System (Beckman Coulter, Brea, CA, USA) by capillary gel electrophoresis according to their nucleotide sizes. The dye signal strength was measured in arbitrary units (A.U.) of optical fluorescence. The data were analyzed using the Fragment Analysis module of the GeXP system software and then transferred to the analysis module of eXpress Profiler software. Normalization was performed with β-actin as reference gene, according to manufacturer’s instructions.

### Statistical analysis

Statistical analysis (n = 3) was conducted by one-way analysis of variance with Tukey’s multiple comparison test using Statistical Package for the Social Sciences (SPSS Inc., Chicago, Illinois, USA) version 21.0 and *p* < 0.05 was considered as significantly different.

## Results

### ORF-protected SH-SY5Y cells against H_2_O_2_-induced neurotoxicity

**Figure 1:**
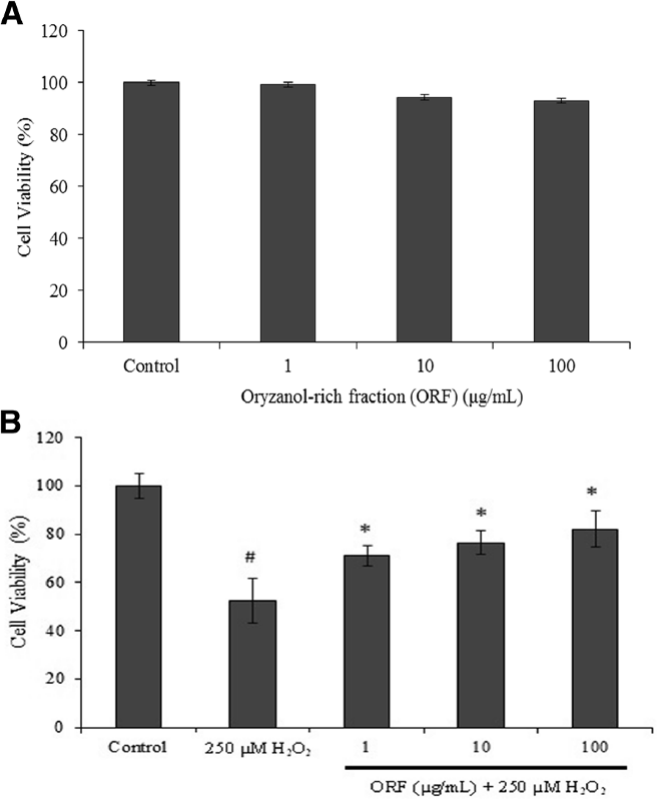
**Cell viability (MTT assay) of SH-SY5Y cells pretreated with Oryzanol-rich fraction (ORF) for 24 h followed by subsequent exposure to 250 μM H**_**2**_ **O**_**2**_ **for 24 h. (A)** ORF alone; **(B)** ORF + 250 μM H_2_O_2_. Results are mean ± SD. ^#^ *p* <0.05 *versus* control, *p < 0.05 *versus* H_2_O_2_.

Treatment of SH-SY5Y cells with ORF (1–100 μg/mL) did not show much toxicity (Figure 1A). However, treatment of the cells with 250 μM H_2_O_2_ for 24 h resulted in significant cell death [24]. When cells were pretreated with ORF for 24 h and subsequently exposed to 250 μM H_2_O_2_ for another 24 h, the resulting toxicities observed were not as significant as with H_2_O_2_ treatment alone (Figure 1B).

### ORF prevented H_2_O_2_-induced morphological changes in SH-SY5Y cells

**Figure 2:**
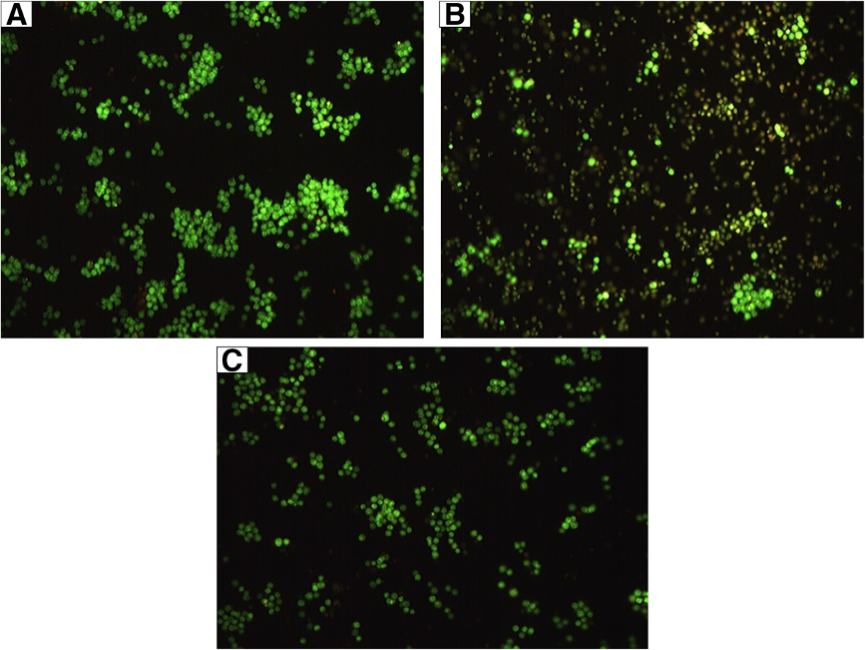
**Acridine orange (AO)–propidium iodide (PI) double staining cell morphological assessment.** Morphological changes in SH-SY5Y cells pretreated with Oryzanol-rich fraction (ORF) for 24 h followed by subsequent exposure to 250 μM H_2_O_2_ for 24 h. **(A)** untreated cells (control); **(B)** 250 μM H_2_O_2_ alone; **(C)** 100 *μ* g/mL ORF +250 μM H_2_O_2_. Viable cells are stained green by acridine orange; Late apoptotic and necrotic cells are stained orange and red by propidium iodide.

AO/PI double staining distinguishes between viable, apoptotic and necrotic cells. Viable cells will normally show round and green nuclei, similar to those of the untreated cells in this study (Figure 2A). Late apoptotic and necrotic cells will stain orange and red as displayed by the H_2_O_2_–treated cells (Figure 2B). In this study, the nuclei of the cells pretreated with ORF stained orange and red but with less intensity that those of H_2_O_2_–treated cells (Figure 2C).

### ORF protected SH-SY5Y cells against H_2_O_2_-induced cell death

**Figure 3:**
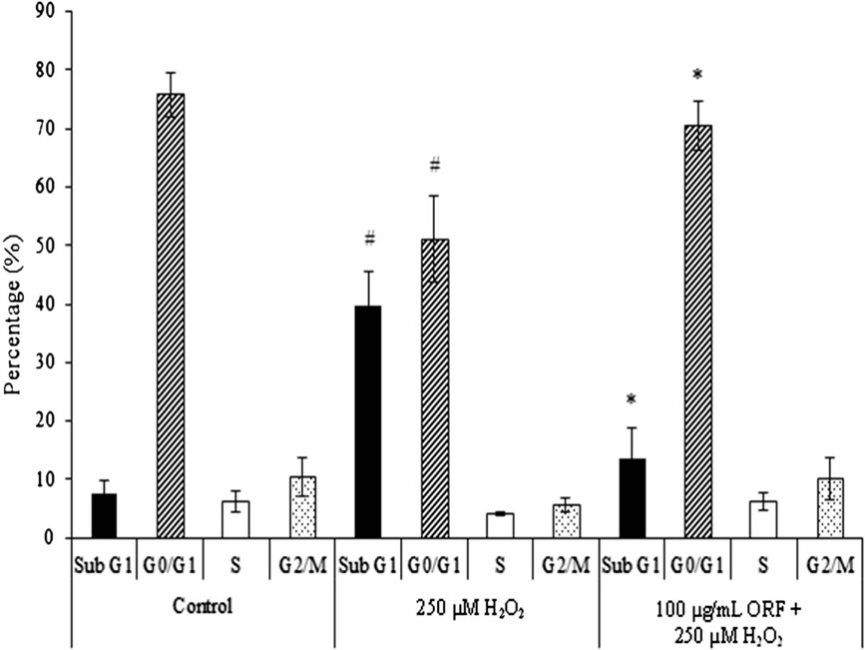
**Cell cycle analysis.** Flow cytometric measurement of cell death and cell cycle on SH-SY5Y cells pretreated with Oryzanol-rich fraction (ORF) (100 μg/mL) before exposure to 250 μM H_2_O_2_ over 24 h. Results are mean ± SD. ^#^ *p* <0.05 *versus* control, *p <0.05 *versus* H_2_O_2_.

Figure 3 showed significant cell death (at Sub G1) (40% ± 5.88%) upon exposure to 250 μM H_2_O_2_ in comparison to untreated cells (8% ± 2.34%), *p* < 0.05. In contrast, pretreatment with 100 μg/mL ORF did not produce as much dead cells (14% ± 5.0%) as with H_2_O_2_ treatment alone, *p* < 0.05. In addition, there was no significant difference in cell populations at S and G2/M phases among control, H_2_O_2_ alone and ORF treatment.

**Figure 4:**
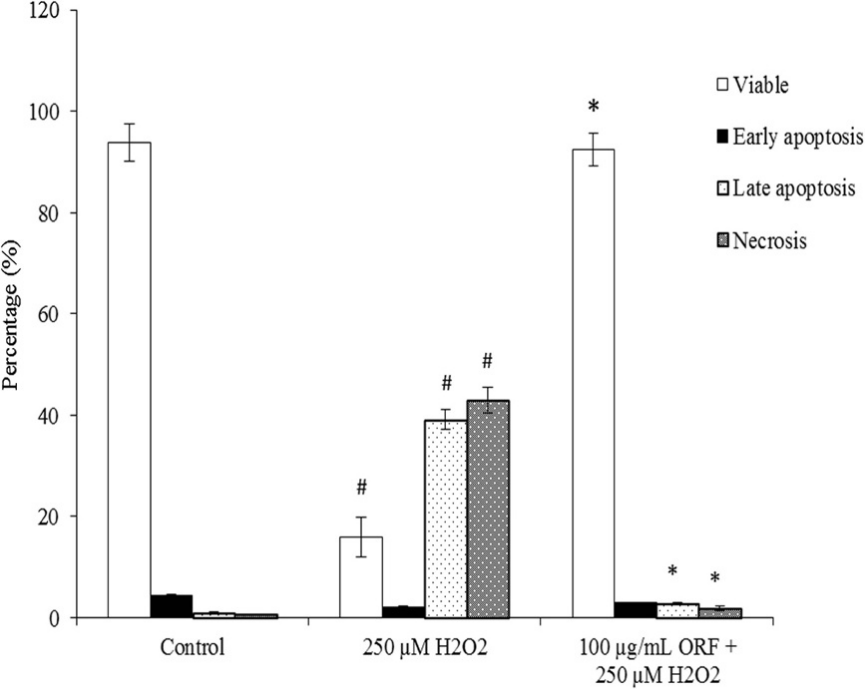
**Annexin V-FITC and propidium iodide staining assay of SH-SY5Y cells following exposure to 250 μM H**_**2**_ **O**_**2**_ **over 24 h, in the presence or absence of 24 h Oryzanol-rich fraction (ORF) (100 μg/mL) pretreatment.** Results are mean ± SD. ^#^ *p* < 0.05 versus control, *p <0.05 versus H_2_O_2_.

Figure 4 showed that exposure of SH-SY5Y cells to H_2_O_2_ demonstrated significant differences in viable, late apoptosis/early necrosis and late necrosis states in comparison to untreated cells. The untreated cells showed 94% ± 3.67% viability, while incubation with 250 μM H_2_O_2_ reduced the cell viability, to only 16% ± 3.97% represented by early apoptosis (2% ± 0.05%), late apoptosis (39% ± 2.2%) and necrosis (43% ± 3.85%). The results indicated that H_2_O_2_ at 250 μM was highly toxic to SH-SY5Y cells. Pretreatment with ORF (100 μg/mL), however, protected the cells against H_2_O_2_-induced apoptosis, as demonstrated by 92.41% ± 3.3% viability, which was similar to the untreated cells (Figure 4).

### Effects of ORF on transcriptomic regulation of apoptotic and antioxidant genes in SH-SY5Y cells exposed to H_2_O_2_

**Figure 5:**
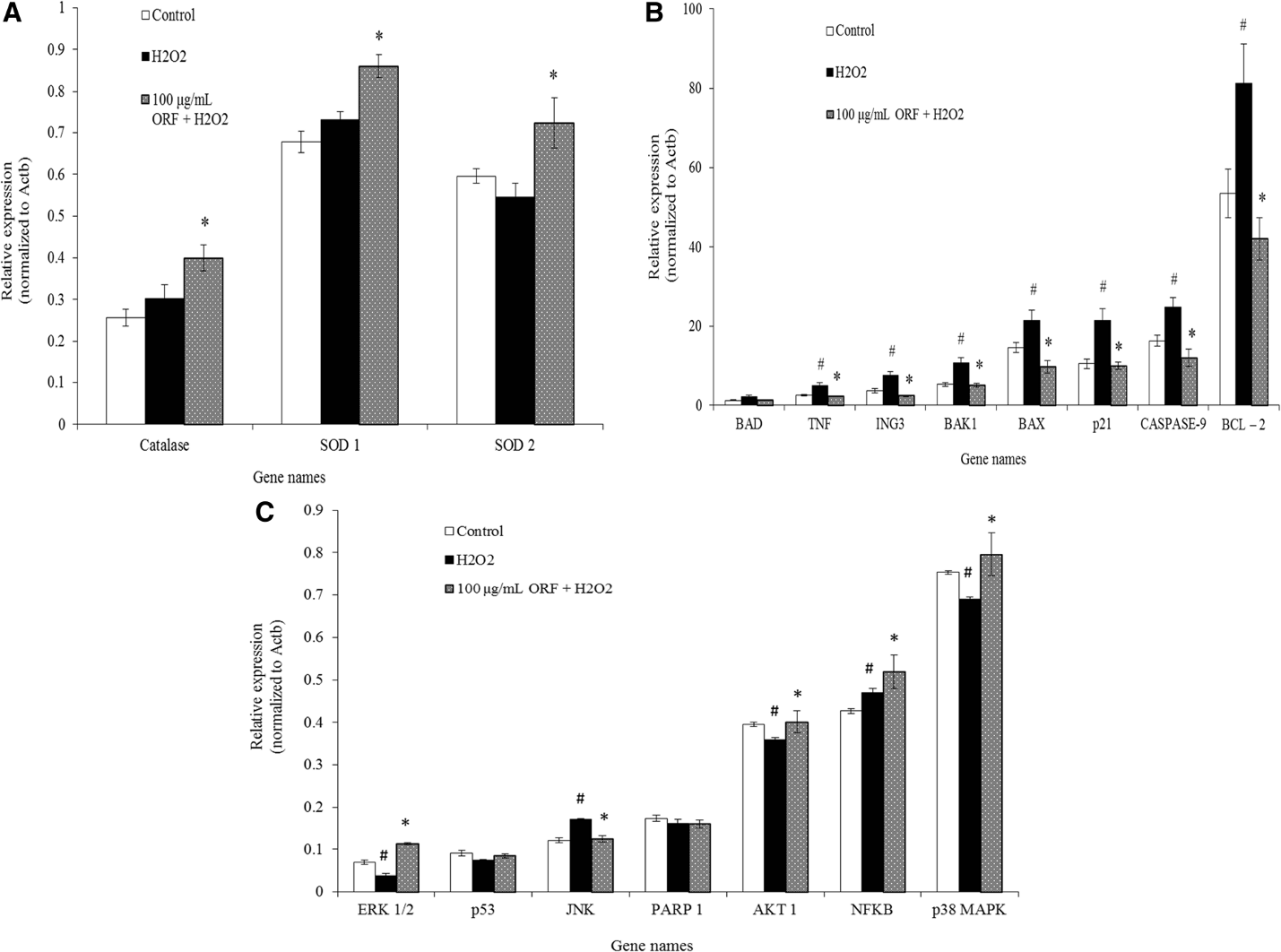
**Expression of (A) Antioxidant genes (catalase, SOD 1 and SOD 2), (B) Downstream apoptotic genes (BAD, TNF, ING3, BAK1, BAX, p21, caspase-9 and BCL-2), and (C) Upstream apoptotic genes (ERK1/2, p53, JNK, PARP1, AKT1, NF-Kβ, and p38), following treatment with 100 μg/mL Oryzanol-rich fraction (ORF) and subsequent exposure with 250 μM H**_**2**_ **O**_**2**_ **.** Results are the mean ± SD. ^#^ *p* < 0.05 versus control, *p <0.05 versus H_2_O_2_.

H_2_O_2_-treatment induced upregulation of antioxidant genes in SH-SY5Y cells in comparison to untreated cells (Figure 5A). Furthermore, pretreatment of SH-SY5Y cells with ORF (100 μg/mL) prior to H_2_O_2_ exposure upregulated catalase, SOD 1 and SOD 2 genes more than with H_2_O_2_ exposure alone or in untreated cells (*p* < 0.05). Additionally, H_2_O_2_ significantly upregulated (*p* < 0.05) the expression levels of TNF, ING3, BAK1, BAX, p21, caspase-9 and BcL-2 genes, but not BAD gene, in comparison to untreated cells (Figure 5B). Pretreatment with ORF (100 μg/mL) resulted in downregulation of the expression levels of TNF, ING3, BAK1, BAX, p21 and caspase-9 genes, (*p* < 0.05). In the presence of H_2_O_2_, SH-SY5Y cells showed significantly upregulated (*p* < 0.05) JNK and NF-Kβ gene expression levels, and downregulated ERK1/2, AKT1 and p38 levels, in comparison to untreated cells. No changes were observed for p53 and PARP1 expression levels (Figure 5C). Pretreatment with ORF (100 μg/mL) upregulated the expression of ERK1/2, PARP1, AKT1 and NF-Kβ genes, (*p* < 0.05), but downregulated that of JNK.

## Discussion

In the present study, the neuroprotective effects of ORF were evaluated against toxicity caused by H_2_O_2_ on differentiated SH-SY5Y cells. Also, to minimize hazards associated with extraction of plant bioresources, a green technology (SFE) was used to extract ORF. In neuronal cells, H_2_O_2_ activates intracellular defence mechanisms including the upregulation of endogenous antioxidants, which are meant to protect cells from damage by H_2_O_2_. However, in excess, apoptosis and cell death ensue when the defence mechanisms fail to counter the effects of the H_2_O_2_[25]. The increasing interest in plant bioresources that can potentiate antioxidant systems is based on the notion that beyond the normal cellular threshold for apoptosis and cell death, such exogenous antioxidants could complement the cellular defence systems in countering H_2_O_2_-induced damage [1, 26, 27]. In this study, cell viability and morphology of SH-SY5Y cells were preserved by ORF in the presence of H_2_O_2_, suggesting that ORF likely was able to alter some pathways leading upto oxidative damage and/or apoptosis. The protective effects of ORF against H_2_O_2_-induced cell death were further corroborated by flow cytometric analyses, which indicated that ORF reduced the apoptotic and necrotic cells’ population in SH-SY5Y cells.

Changes in the expression of antioxidant genes in the present study due to H_2_O_2_ are similar to what we have reported previously [28]. These changes suggested activation of antioxidant defence systems in SH-SY5Y cells to counter H_2_O_2_. The presence of ORF potentiated the expression of the antioxidant genes, suggesting further protection for the cells. As can be recalled, a very close link exists between pathways leading up to cell survival and those that mediate apoptosis and cell death due to oxidative damage. MAPKs and Akt are believed to be involved in these processes including cell growth, survival, differentiation and apoptosis responses [29]. Oxidative stress is reported to induce apoptotic cell death through activation of transcriptional factors such as MAPKs [25], and especially JNK [30, 31], which is known to be involved in pro-apoptotic signaling [32]. JNK activation facilitates the decrease of mitochondrial membrane potential followed by release of cytochrome c, which then activates caspase-9 and caspase-3, eventually leading to cell death [33]. Our results showed that H_2_O_2_ induced JNK and caspase-9 activation, which were both attenuated by ORF treatment, suggesting that the protective effects of ORF against H_2_O_2_-induced injury in SH-SY5Y cells were partly mediated through its protection of the mitochondria.

Activation of ERK due to oxidative stress is reportedly mediated by growth factor receptors [34–36]. When growth factor receptors undergo phosphorylation in response to oxidative insults such as H_2_O_2_, the resulting changes attenuate ERK activation. Similarly, expression of inactive mutant forms of various growth factor receptors reduces activation of ERK by oxidative stress [34], while over expression of certain normal growth factor receptors in rat PC12 cells exposed to H_2_O_2_, results in enhanced activation of ERK [37]. Observations like these have given rise to suggestions of ERK activation as a survival factor following oxidative injury [38, 39]. In the present study, H_2_O_2_ downregulated ERK1/2 gene expression, while ORF pretreatment resulted in upregulation of the gene. This suggested that in response to H_2_O_2_-induced oxidative stress, ORF pretreatment may trigger the expression of growth factor receptors in SH-SY5Y cells leading to activation of ERK1/2 gene as a protective mechanism. In addition, p38 MAPK is known to be a stress kinase, and its activation may lead to cell death. However, while this assumption is correct in most cases, cause-effect studies have also found that activation of p38 MAPK by stress stimuli may not necessarily promote death, but sometimes could enhances cell survival and DNA repair [40]. Similarly, activation of Akt in response to oxidant exposure appears to be mediated through growth factor receptors also [41]. In SH-SY5Y cells, Akt activation is linked to inhibition of apoptosis especially in the presence of oxidative stress [42, 43]. In the present study, upregulation of Akt by ORF pretreatment suggested that ORF was anti-apoptotic.

Dysregulation of TNF production has been implicated in a variety of human diseases. Binding of TNF to its receptor may result in activation of NF-κB, activation of MAPK pathways or induction of death signaling. NF-κB is a heterodimeric transcription factor and translocates to the nucleus to mediate the transcription of a vast array of proteins involved in cell survival and proliferation, inflammatory response and anti-apoptotic factors. ORF pretreatment enhanced the gene expression of NF-κB, indicating that NF-κB signaling pathway was likely involved in promoting survival and anti-apoptosis in SH-SY5Y in the presence of oxidative damage.

Oxidative stress often leads to cell death through apoptosis, and in the present study, activation of the genes encoding the Bcl-2 family proteins (i.e. Bcl-2, Bak1 and Bax) and caspase-9 suggested the activation of apoptosis in the cells. Caspase-9 is thought to activate caspase-3, which is known to cleave many nuclear DNA repair enzymes, such as PARP, resulting in nuclear DNA damage and apoptosis [3]. Moreover, ING3 overexpression was also reported to have induced apoptosis in RKO cells, through activation of p21 and Bax [44]. Upregulation of ING3 in the present study due to H_2_O_2_, therefore, also suggested induction of apoptosis in the cells. Interestingly, ORF pretreatment resulted in downregulation of Bcl-2, Bak1, Bax, p21, ING3 and caspase-9, indicating a tendency for anti-apoptosis.

**Figure 6:**
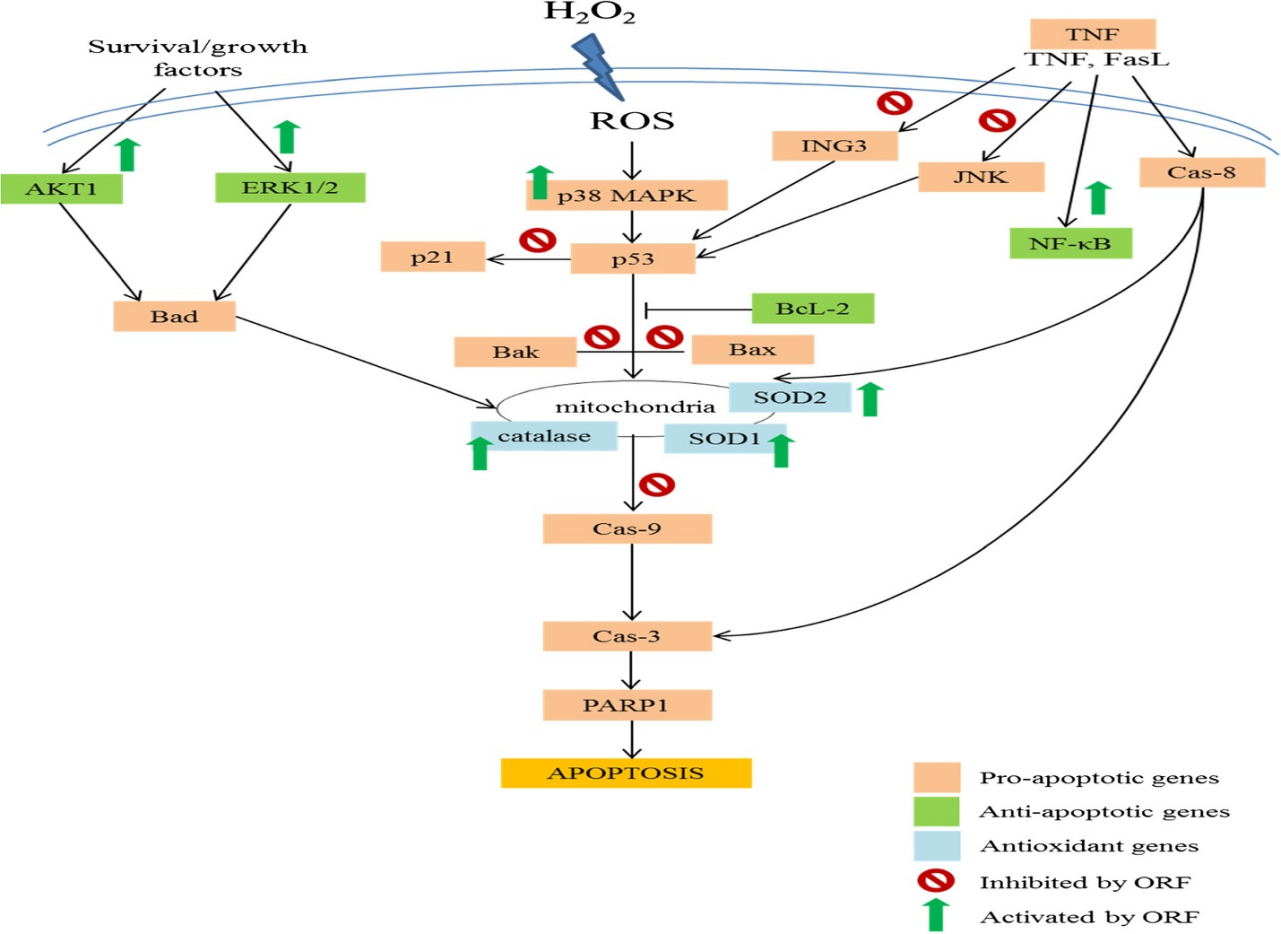
**Schematic presentation of the proposed mechanistic basis for the neuroprotective effects of ORF against H**_**2**_ **O**_**2**_ **-induced neurotoxicity in human differentiated SH-SY5Y cells.** Oxidative stress likely activates pro-apoptotic genes (i.e. JNK, TNF, ING3, BAK1, BAX, p21 and caspase-9) and downregulates anti-apoptotic genes (i.e. ERK1/2, AKT1 and NF-Kβ), resulting in cellular apoptosis. In contrast, ORF likely upregulate endogenous antioxidant defences (i.e. Catalase, SOD1 and SOD2) that can enhance cell survival. Additionally, ORF may downregulate pro-apoptotic genes thereby preventing mitochondrial malfunction and caspase activation. Activation of anti-apoptotic genes likely also contributes to enhanced cell survival.

Taken together, ORF was found to protect SH-SY5Y cells against H_2_O_2_-induced neurotoxicity possibly through multi-signaling pathways (Figure 6). The protective effects of ORF on SH-SY5Y cells were likely mediated through upregulation of antioxidant genes (catalase, SOD 1 and SOD 2), downregulation of pro-apoptotic genes (JNK, TNF, ING3, BAK1, BAX, p21 and caspase-9), and upregulation of anti-apoptotic genes (ERK1/2, AKT1 and NF-Kβ). The findings from this study suggest that rice bran could potentially be a source of antioxidants that may have huge implications on the study and management of neurodegerative diseases.

## Conclusions

In this study, ORF protected SH-SY5Y cells against H_2_O_2_-induced neurotoxicity as evidenced by the reduced cytotoxicity, inhibition of apoptosis, and gene expression changes (upregulation of antioxidant genes, downregulation of pro-apoptotic genes, and upregulation of anti-apoptotic genes) that tended towards cell survival. The results suggested that ORF could protect SH-SY5Y cells against oxidative stress-mediated apoptosis. These findings could have huge implications on future studies on the potential use of ORF in managing neurodegenerative diseases caused by oxidative injury.

## Abbreviations

AKT- v: Akt murine thymoma viral oncogene
AO-PI: Acridine orange–propidium iodide
BAD: Bcl-2-associated death promoter
BAK1: BCL2-antagonist/killer 1
BAX: Bcl-2-associated X
ERK: Extracellular regulated kinase
H_2_O_2_: Hydrogen peroxide
ING3: Inhibitor of growth 3
JNK- c: Jun N-terminal kinase
MAPK: Mitogen-activated protein kinases
MTT: (3-[4,5-dimethylthiazol-2-yl]-2,5-diphenyl-tetrazolium bromide)
NF-KB: Nuclear factor kappa B
ORF: Oryzanol-rich fraction
P13K: Phosphoinositide 3-kinase
p21: Tumor suppressor gene 21
ROS: Reactive oxygen species
SFE: Supercritical carbon dioxide fluid extraction
SOD: Superoxide dismutase
TNF: Tumor necrosis factor.
